# The Courage to Be Kind: Leadership, Civility and the Culture of Care

**DOI:** 10.1111/ans.70523

**Published:** 2026-02-06

**Authors:** Carlton Irving

**Affiliations:** ^1^ New Zealand Graduate School of Medicine The University of Waikato Te Whare Wānanga o Waikato Hamilton New Zealand

Kindness in healthcare is not a luxury; it is a clinical necessity. Far from being a soft skill, it demands discipline, especially in high‐pressure or hostile environments. In Aotearoa New Zealand, kindness is not a courtesy but a clinical discipline and form of leadership. When Māori whānau encounter hostility, they avoid returning to care, whereas services grounded in manaaki (care and hospitality) and whanaungatanga (relational connection) keep people engaged [1] practising that consistently takes courage.

In modern healthcare, kindness is often undervalued, perceived as secondary to technical competence. Yet kindness, understood as a deliberate act of respect and generosity, has measurable effects on patient safety, diagnostic accuracy and team morale [2, 3, 4]. Leadership that centres relational values is associated with improved team cohesion and patient experience, as shown in organisational research on wellbeing and culture [5]. In surgery, where coordination, pace and hierarchy are intense, small acts of civility shape team performance and patient outcomes.

Kindness under pressure is not instinctive; it is chosen. Leaders, particularly clinicians, shape workplace culture not by slogans but through micro behaviours. Macklin and colleagues found that kindness, when consciously practised, enhances compassion and empathy and reduces moral distress in clinical teams [2]. When leaders are civil, others mirror that behaviour. Structured interventions to build civility competencies have been associated with reductions in incivility and stronger collegial trust [6]. Mātauranga Māori (māori knowledge) reminds us values such as manaaki (care and hospitality) and kotahitanga (unity of purpose) are not abstract ideals but methods for sustaining relationships under stress. To live them requires conscious effort, especially when exhausted or confronted with hostility.

There are three rules we can use to practise kindness.

The first rule is simple. Be kind.

Choose actions that reduce harm, not add to it. Between stimulus and response there is choice of how we will behave, and those moments define us. We live in a world saturated with social media, where grace is thinning. A glance at any comments section shows the bilge. Viktor Frankl wrote that ‘everything can be taken from a person but one thing—the freedom to choose one's attitude in any circumstance, to choose one's own way’ [7]. In healthcare those moments arrive constantly. A tone of voice. A look. A hurried word. Letting emotion drive can normalise incivility. The quiet kind. Dismissiveness. Side comments. Leaving people out.

Civility, as Clark reminds us, is a clinical competency, not a personality trait [6]. It builds effective teamwork, vital in theatre environments, reduces staff turnover, and builds psychological safety.

Effective teamwork improves patient safety, and it begins the moment a team forms. An authentic briefing at the start of an operating list, with a leader who listens and responds constructively, signals that every voice matters. When concerns are acknowledged and acted on, team members are more willing to speak up again, and in theatre that repeated willingness to speak is essential for patient safety, as described in recent work on responses to speaking up in the operating room [8].

In short, do the basics well. Say a colleague's name correctly. Thank people in public. Offer performance feedback in private. Respect can be taught, practised and shared. If we can perform complex procedures under pressure, we can manage basic civility in theatre or ward rounds.

The second rule: Put the shopping trolley back.

We all know the irritation at the supermarket of trolleys left askew across the car park. In healthcare our trolleys are open loops. Labs not checked, referrals not sent, families not updated. Each small omission adds risk. Putting the trolley back means closing the loop. Put your name on your theatre hat and practise closed loop communication so everyone knows who is speaking and what is needed, because clear names and clear checks lift teamwork and keep patients safe [9]. Write the post‐op discharge script, chart the regular medication post‐operatively. Call the whānau (family/contact people) when plans change or the operation is complete to let them know how it has gone. Make sure the rural patient who travelled for hours feels seen. Thank people by name and mean it, from junior doctors to theatre nurses and anaesthetists [10].

These are acts of kotahitanga (unity of purpose) that say I have your back. Culture is not built by policy lines. It is built by repeatable, responsible behaviour. Evidence shows that consistent prosocial behaviour and clear communication reduce risk and improve retention [11]. Small acts of care, done reliably, grow into systems of safety.

The third rule: Have a backbone.

Kindness without courage is decoration. Leadership means modelling good behaviour but also calling out poor behaviour, kindly, firmly, and early. When rules one or two are broken, silence becomes complicity. RACS has promoted a culture of respect, often referencing the phrase ‘the standard you walk past is the standard you accept’ [12, 13]. A just culture forgives but does not excuse. Parkinson describes this as the heart of restorative practice: naming transgressions early, not to punish but to protect relationships [14]. Having a backbone might mean raising a concern to safeguard a patient, supporting a colleague who challenges bias, or calmly confronting rudeness from a senior. True courage in leadership is rarely loud; it is quiet consistency, doing the right thing when no one is watching.

Kindness therefore does not mean avoiding boundaries. There are moments when clarity, firmness or urgent action must lead. Kindness needs judgement. It sits alongside accountability, clinical precision and time pressure. It guides behaviour but does not replace the discipline of good medicine.

Kindness has a growing evidence base. Rudeness impairs diagnostic reasoning and team performance [3]; incivility contributes to burnout, communication failures and turnover [11, 15]. Conversely, gratitude and civility enhance collaboration and collective efficacy [4]. Compassion and kindness can activate neural reward circuits that buffer clinicians against emotional exhaustion [5]. Within Aotearoa New Zealand, values such as Manaaki (care and hospitality) and whanaungatanga (relational connection) strengthen resilience and cohesion under pressure [14]. In multicultural teams, leaders who cultivate cross‐cultural empathy reduce conflict and improve care outcomes [16]. Matthews and Govindasamy note that compassion in medicine protects both staff and patients from moral injury [17].

Kindness, then, is not a soft trait but a safety practice. It stabilises teams and sustains clinicians. It can be taught, measured and scaled. To lead with manaaki (respect and generosity) is to choose care, to be kind even when it is inconvenient, to put the shopping trolley back when no one is watching, and to have a backbone when boundaries must be drawn. Culture lives in what we tolerate and what we model.

The courage to be kind is the courage to lead well.

## Author Contributions

**Carlton Irving:** conceptualization, investigation, writing – review and editing, writing – original draft.

## Data Availability

Research data are not shared.
